# Transforming primary care: improving on the medical home model

**DOI:** 10.3109/13561820.2011.571316

**Published:** 2011-05-09

**Authors:** Linnea Windel, Laura Anderko, Tamara Konetzka

**Affiliations:** 1 VNA Health Care, Aurora, USA; 2 Department of Nursing & Health Studies, Georgetown University, Washington, USA; 3 Department of Health Studies, University of Chicago, Chicago, USA

## INTRODUCTION

Given the convergence of exponential spending on health care in the US, poor health outcomes relative to other countries, an aging population, and outdated methods of delivering primary care, there is no better time for instituting a “disruptive” innovation in primary care redesign than the present (Christensen, Bohmer, & Kenagy, 2000). In the midst of health reform in the US, the concept of the patient-centered medical home (PCMH) is garnering significant attention as a potential solution to address financial and performance issues (Vincent & Velkoff, 2010). While the model has worthy attributes, including an emphasis on care coordination and patient engagement, recent studies highlight the gaps and limitations in the existing approaches (Nutting et al., 2009; Rogers, 2008). Some of these gaps and limitations in the traditional PCMH model include difficulties in accomplishing shared decision making with the patient and health care team, effective communication across services, and offering a broad range of health care services.

The authors propose an alternative, primary care redesign model intended to build on the strengths of the PCMH model and address some of the current shortcomings in implementation. This alternative model is designed to improve patient health and satisfaction, while decreasing costs and may be particularly effective among the frail elderly.

## THE PATIENT CENTERED MEDICAL HOME MODEL

Although considered by many to be a new idea, the medical home concept was first introduced in 1967. A review of the principles, standards, position papers, and projects to date reveals several themes required of a PCMH. Basic criteria include (a) directly provided services, (b) physician coordination of care, (c) patient involvement in planning care, and (d) collecting, sharing, and monitoring data to improve care.

Limitations in how this model is operationalized have been highlighted in recent research (Nutting et al., 2009; Sinsky, Sinksy, Althaus, Tranei, & Thiltgen, 2010). Directly provided services in current PCMH models are typically limited to primary care and laboratory testing. Second, a PCMH requires new skills for many physicians in care coordination and management and working in teams with shared decision making. Finally, most PCMH practices do little to assess the home and community factors affecting the patient's health (American College of Physicians, 2005; Fields, Leshen, & Patel, 2010; Nutting et al., 2009; Rogers, 2008).

However, recent research findings suggest that a health care system of higher quality at lower cost will require: easy access, a wide array of services offered by the primary care facility, care provided over sustained periods of time, care that is coordinated amongst various disciplines/services, and patient-focused care with an orientation to family and community needs (Fields et al., 2010; Nutting et al., 2009).

Although PCMH models are beginning to emerge that address some of these requirements, none to date has been able to incorporate all of these within one model. The authors propose an alternative primary care delivery model that is consistent with higher quality/lower cost health system research findings and builds on the strengths of the PCMH model, while striving to overcome its implementation challenges.

## AN ALTERNATIVE MODEL OF PRIMARY CARE

*Care Plus*, an alternative to the traditional PCMH model, is defined as a “patient-centered health care home” model that includes a wide array of directly provided primary care and support services, coordination by a community health registered nurse (RN) (rather than a physician), and regular RN assessment visits in the patient home.

### Broad range of services

First, patients have access to a broad range of directly provided services in which the challenges of effective coordination and communication are minimized from the outset. Community-based organizations (such as Community Health Centers) are one such platform that may logically serve as an effective base for this new model of primary care delivery. Consistent with the proposed tenets of the model, this depth and breadth of directly provided services is considerably different from the typical PCMH model and particularly important to an older population who suffer from complex, chronic health conditions. See Table I.

**Table I:** Comparison of patient-centered primary care delivery models.

Services	Traditional PCMH	Care Plus model
Primary care, office-based	X	X
Primary care, home-based		X
Nurse-led care management		X
Physician-led care management	X	
Ongoing, in-home assessments by nurse care manager		X
Community-health perspectives		X
Medicare-certified home health		X
Mental health		X
Nutrition		X
Clinical pharmacy		X
Mammography		X
Social work services		X
Telemonitoring		X
Laboratory testing and tracking	X	X
Medication monitoring	X	X
Specialty care referrals and coordination	X	X
Electronic record management	X	X
Interdisciplinary team approach	X	X
Quality improvement system	X	X
Chronic disease management	X	X
Manage hospital transitions	X	X
A focus on patient satisfaction	X	X
Shared decision making with patient and family	X	X
Ongoing service provision	X	X

### Community health RN as care manager

Second, this model positions a community health RN as the care manager, capitalizing on skills inherent in this specialty such as building collaborative partnerships and resource management. The RN Care Manager directs the care, coordinates it, and facilitates the communication among everyone involved. While the PCMH concept places coordination activities with the physician (American College of Physicians, 2005), we believe the skills inherent in *“community health”* nursing - the ability to navigate the system and coordinate services and information for better client outcomes - are precisely the skills needed to improve client outcomes in the complex and fragmented primary health care system that exists today. The RN Care Managers coordinate care for older adult patients while using home environment, family, and community information to develop the individual plan of care.

### Ongoing home assessments

Finally, in contrast to the PCMH model, *Care Plus* emphasizes the importance of regularly assessing the patient in his or her own home in order to more fully understand the patient problems and needs and use that information to create an improved patient plan of care. Issues that are especially relevant to the older population including patient safety, functional status, medication issues, nutritional status, and social support are markedly sensitive to assessment in the home. See Table I for model comparisons.

### Determining the impact of this model

A randomized clinical trial to study the effectiveness of the proposed model of primary care will begin in early 2011. The patient population is expected to have a heavy disease burden with chronic illnesses including diabetes, congestive heart failure, and coronary artery disease, among others. Specific outcomes to be studied include: patient satisfaction, self-rated health, functional status, and cost outcomes including pre- and post- intervention hospitalizations and nursing home stays.

## CONCLUDING COMMENTS

Never has innovation, for the purpose of improving health, patient satisfaction, and decreasing cost, been more important. Incorporation of community nursing into primary care delivery may provide the solutions for improving health, satisfaction, and reducing costs.
